# Coding Properties of Three Intrinsically Distinct Retinal Ganglion Cells under Periodic Stimuli: A Computational Study

**DOI:** 10.3389/fncom.2016.00102

**Published:** 2016-09-23

**Authors:** Lei Wang, Yi-Hong Qiu, Yanjun Zeng

**Affiliations:** ^1^Neuroscience and Intelligent Media Institute, Communication University of ChinaBeijing, China; ^2^School of Biomedical Engineering, Shanghai Jiao Tong UniversityShanghai, China; ^3^Biomedical Engineering Center, Beijing University of TechnologyBeijing, China

**Keywords:** retinal ganglion cell, morris-lecar neuron, spike-rate, spike-latency, periodic stimuli

## Abstract

As the sole output neurons in the retina, ganglion cells play significant roles in transforming visual information into spike trains, and then transmitting them to the higher visual centers. However, coding strategies that retinal ganglion cells (RGCs) adopt to accomplish these processes are not completely clear yet. To clarify these issues, we investigate the coding properties of three types of RGCs (repetitive spiking, tonic firing, and phasic firing) by two different measures (spike-rate and spike-latency). Model results show that for periodic stimuli, repetitive spiking RGC and tonic RGC exhibit similar spike-rate patterns. Their spike- rates decrease gradually with increased stimulus frequency, moreover, variation of stimulus amplitude would change the two RGCs' spike-rate patterns. For phasic RGC, it activates strongly at medium levels of frequency when the stimulus amplitude is low. While if high stimulus amplitude is applied, phasic RGC switches to respond strongly at low frequencies. These results suggest that stimulus amplitude is a prominent factor in regulating RGCs in encoding periodic signals. Similar conclusions can be drawn when analyzes spike-latency patterns of the three RGCs. More importantly, the above phenomena can be accurately reproduced by Hodgkin's three classes of neurons, indicating that RGCs can perform the typical three classes of firing dynamics, depending on the distinctions of ion channel densities. Consequently, model results from the three RGCs may be not specific, but can also applicable to neurons in other brain regions which exhibit part(s) or all of the Hodgkin's three excitabilities.

## Introduction

In the vertebrate retinas, ganglion cells are the principal neurons, through which visual information is effectively processed and then reliably transmitted to the lateral geniculate nucleus (LGN) and the visual cortex (Kandel et al., 2000). Thus, the capability of retinal ganglion cells (RGCs) in encoding incoming stimulus becomes extremely important. Previous studies suggested that RGCs can be classified into several different types based on their response patterns, e.g., repetitive spiking (Fohlmeister and Miller, 1997; Margolis and Detwiler, 2007; Fohlmeister et al., 2010), tonic firing (Wang et al., 1997; Tsai et al., 2011), phasic firing (Kawai and Sterling, 2002; Tabata and Kano, 2002), and adapting activity (Kim and Rieke, 2003; Li et al., 2012; Xiao et al., 2013a,b). These different firing behaviors may manifest different coding manners that RGCs adopt in processing neural signals.

It has been accepted that neural information can be encoded in a variety of manners, among which rate coding and time coding are the two most commonly used (Dayan and Abbott, 2001). Generally, rate coding refers to the number of spikes within a time window or cycle (Descalzo et al., 2005; van Brederode and Berger, 2008), while time coding refers to the first-spike latency of neural spikes (Chase and Young, 2007) or regularity or sparseness of population spike trains (Mainen and Sejnowski, 1995; Zhang et al., 2010). Several reports have revealed that RGCs can encode incoming signals effectively using these two strategies (van Rullen and Thorpe, 2001; Gollisch and Meister, 2008; Gütig et al., 2013). However, response patterns that RGCs produce under periodic stimuli are not completely clear yet, which need to be figured out.

Previously, Hodgkin classified neurons into three basic classes based on their firing frequency vs. stimulus intensity (*f-I*) curves (Hodgkin, 1948). Generally, Class I neurons fire slowly in response to weak stimulus, and display a continuous *f-I* curve; while class II neurons exhibit a discontinuous *f-I* curve for their inability to produce spikes below certain threshold intensities. Class III neurons, however, fail to spike repetitively, and typically spike only once at the onset of stimulus (Izhikevich, 2007; Prescott et al., 2008a; Wang et al., 2013). Functional roles of the three classes of neurons have been extensively investigated during the past decades (Marella and Ermentrout, 2008; Bogaard et al., 2009; Fink et al., 2011). For instance, it has been proposed that class I neurons act as integrators or coincidence detectors, while class II neurons act as resonators (Izhikevich, 2000). For time-varying inputs, class III neurons serve as slope detectors or band-pass filters (Gai et al., 2010).

In this study, we firstly investigate how RGCs react to periodic stimuli through spike-rate coding and spike-latency coding. Ionic models which can characterize the electrophysiological properties of three RGCs (repetitive spiking, tonic firing, and phasic firing) are introduced. Simulation results demonstrate that diverse response patterns can be observed in RGCs, not only within the same types, but also among different types. In addition, stimulus amplitude plays a conspicuous role in transforming the response patterns from one type to another. Finally, we show that RGCs can exhibit all the Hodgkin's three classes of firing dynamics by only adjusting the conductances of several ion channels.

## Models and methods

### RGC model

Ionic model of RGCs with repetitive spiking was adopted from Fohlmeister and Miller (1997). The model had only one-compartment, which represents the soma, with five voltage-gated ion channels and a leak channel (*I*_*L*_) distributed on the soma. The five ion channels were: inactivating sodium (*I*_*Na*_), delayed-rectifier potassium (*I*_*K*_), calcium (*I*_*Ca*_), A-type potassium (*I*_*A*_), and calcium-activated potassium (*I*_*KCa*_) channels. Later, to simulate the tonic and phasic firings in RGCs, a modified RGC model was introduced (Wang et al., 2014). The modified model retained the five voltage-gated ion channels, but revised the inactivating sodium channel by adding two slow variables. Numerical results showed that the model can well reproduce the tonic and phasic firing behaviors by adjusting several parameters. Adapting activity is another typical firing behavior observed in RGCs, due to the lack of proper ion channels and corresponding neuron models, we did not consider adapting activity in the present study.

Mathematical description in Equation (1) represents the common model of RGCs (Fohlmeister and Miller, 1997).

(1) $$\begin{array}{l} C_{m}\frac{dV}{dt}=-I_{Na}-I_{K}-I_{Ca}-I_{A}-I_{KCa}-I_{L}+I \end{array}$$

where *C*_*m*_ is the specific membrane capacitance, *V* is the membrane potential, and *I* is the stimulus applied to the neuron. In this study, *I* = *I*_*amp*_sin(2π*ft*) is used to mimic the external periodic input, where *I*_*amp*_ is the stimulus amplitude, *f* is the stimulus frequency. To avoid the model neurons overly hyperpolarized, we set the negative values of *I* to zero, the value under which the model neurons are quiescent.

For repetitive spiking RGC, $I_{Na}=g_{Na}m^{3}h\left(V-V_{Na}\right)$, while for tonic and phasic RGC, $I_{Na}=g_{Na}m^{3}hs_{1}s_{2}\left(V-V_{Na}\right)$. The distinction between tonic and phasic RGCs is that they possess different combinational values of sodium channel conductance and potassium channel conductance (Wang et al., 2014).

Specific parameters used in our simulation are given in Table 1, and detailed expressions and gating variables for each ion current are provided in Table 2. In the table, *g*_*Na*_, *g*_*K*_, *g*_*Ca*_, *g*_*A*_, *g*_*KCa*_, and *g*_*L*_ are the conductances for each currents. *V*_*Na*_, *V*_*K*_, and *V*_*L*_ are the reversal potentials. *m, h, s*_1_, *s*_2_, *n, c, a*, and *b* are the gating variables.

**Table 1:** **Specific parameters used in simulations**.

**RGC**		**ML**
*C_m_* = 1 *μ F/cm*^2^	*V_Na_* = 35 *mV V_K_* = −75 *mV*	*C_m_* = 2 *μ F/cm*^2^
*g_Na_* = 50 *mS/cm*^2^ *(Repetitive spiking)*	*V_L_* = −65 *mV T* = 295 *K*	*g_Na_* = 20 *mS/cm^2^ g_K_* = 20 *mS/cm*^2^
= 200 *mS/cm*^2^ *(Tonic firing)*	*R* = 8.314 J/(M· K)	*g_L_* = 2 *mS/cm^2^ V_Na_* = 50 *mV*
= 120 *mS/cm*^2^ *(Phasic firing)*	*F* = 96485 *C/M*	*V_K_* = −100 *mV V_L_* = −70 *mV*
*g_K_* = 12 *mS/cm*^2^ *(Repetitive spiking)*	*r* = 22 *μ m τ _Ca_* = 1.5 *ms*	*β _m_* = −1.2 *mV Φ* = 0.15
= 12 *mS/cm*^2^ *(Tonic firing)*	[*Ca*^2+^]*_diss_* = 0.001 *mM/dm*^3^	*γ _m_* = 18 *mV γ_w_* = 10 *mV*
= 110 *mS/cm*^2^ *(Phasic firing)*	[*Ca*^2+^]*_e_* = 1.8 *mM*	*β _w_* = 0 *mV (Class I)*
*g_Ca_* = 2.2 *mS/cm*^2^ *g_A_* = 36 *mS/cm*^2^	[*Ca*^2+^]*_res_* = 0.001 *mM*	*β _w_* = −13 *mV (Class II)*
*g_KCa_* = 0.05 *mS/cm*^2^ *g_L_* = 0.05 *mS/cm*^2^		*β _w_* = −23 *mV (Class III)*

**Table 2:** **Specific expressions of ion currents and the corresponding gating variables**.

**Currents**	**Gating variables**		
*I_Na_* = *g_Na_m*^3^*hs*_1_*s*_2_(*V*−*V_Na_*)	$\alpha_{m}=\frac{-0.1\left(V+30\right)}{\text{exp}\left(-\left(V+30\right)/10\right)-1}$	$\beta_{h}=\frac{1}{1+\text{exp}\left(-\left(V+20\right)/10\right)}$	$\beta s_{1}=\frac{0.0014}{1+\text{exp}\left(-\left(V+47\right)/4.7\right)}$
	β_*m*_ = 4exp(−(*V*+55)/18)	α_*h*_ = 0.07exp(−(*V*+50)/20)	α_*s*_1__ = 0.00034exp(−*V*/63)
	α_*s*_2__ = 0.0008exp(−*V*/36)		
*I_K_* = *g_K_n*^4^(*V*−*V_K_*)	$\alpha_{n}=\frac{0.02\left(V+40\right)}{1-\text{exp}\left(-\left(V+40\right)/10\right)}$	β_*n*_ = 0.4exp(−(*V*+50)/80)	
*I_Ca_* = *g_Ca_c*^3^(*V*−*V_Ca_*)	$\alpha_{c}=\frac{0.3\left(V+13\right)}{1-\text{exp}\left(-\left(V+13\right)/10\right)}$	β_*c*_ = 10exp(−(*V*+38)/18)	
*I_A_* = *g_A_a*^3^*b*(*V*−*V_K_*)	$\alpha_{a}=\frac{0.006\left(V+90\right)}{1-\text{exp}\left(-\left(V+90\right)/10\right)}$	$\beta_{b}=\frac{0.6}{1+\text{exp}\left(-\left(V+40\right)/10\right)}$	
	α_*b*_ = 0.04exp(−(*V*+70)/20)	β_*a*_ = 0.1exp(−(*V*+30)/10)	
$I_{KCa}=g_{KCa}\frac{{\left([Ca^{2+}]/{\left[Ca^{2+}\right]}_{diss}\right)}^{2}}{1+{\left([Ca^{2+}]/{\left[Ca^{2+}\right]}_{diss}\right)}^{2}}(V-V_{K})$	*IL* = *gL*(*V*−*VL*)		

*In modeling the repetitive spiking behavior, s_1_ and s_2_ are both 1, same as in Fohlmeister and Miller (1997)*.

In contrast to sodium and potassium channels, which have constant values of reversal potential, the value of calcium channel is time-dependent (Equation 2).

(2) $$\begin{array}{l} V_{Ca}=\frac{RT}{ZF}\text{ln }\left(\frac{{\left[Ca^{2+}\right]}_{e}}{{\left[Ca^{2+}\right]}_{i}\left(t\right)}\right) \end{array}$$

where *R* is the gas constant, *T* is the temperature in Kelvin, *Z* is the ionic valency, *F* is the Faraday constant, [*Ca*^2+^]_*e*_ is the concentration of extracellular calcium, and the variation of intracellular calcium concentration [*Ca*^2+^]_*i*_ obeys the Equation (3).

(3) $$\begin{array}{l} \frac{d{\left[Ca^{2+}\right]}_{i}}{dt}=\frac{-5I_{Ca}}{Fr}-\frac{{\left[Ca^{2+}\right]}_{i}-{\left[Ca^{2+}\right]}_{res}}{\tau_{Ca}} \end{array}$$

where *r* is the depth of the shell beneath the membrane for the calcium pump, τ_*Ca*_ is the time constant for calcium current, [*Ca*^2+^]_*res*_ is the free intracellular concentration of calcium ions, and [*Ca*^2+^]_*diss*_ is the calcium dissociation constants (see *I*_*KCa*_ in Table 2).

### Morris-lecar model

For comparison, the Morris-Lecar (ML) model was employed. It has been shown that the ML model can generate the three classes of excitability by only adjusting one parameter (Prescott et al., 2008a; Wang et al., 2013). Moreover, due to its simplicity and accuracy in mimicking the three classes of excitability, ML model has been widely used in analyzing the firing behavior of single neurons (St-Hilaire and Longtin, 2004; Prescott and Sejnowski, 2008; Wang et al., 2011) and population activity of neuronal networks (Marella and Ermentrout, 2008; Fink et al., 2011).

Mathematical expressions for the ML model are in Equations (4) and (5).

(4) $$\begin{array}{r} C_{m}\frac{dV}{dt}=-g_{Na}m_{\infty }\left(V-V_{Na}\right)-g_{K}w\left(V-V_{K}\right)\\ -g_{L}\left(V-V_{L}\right)+I \end{array}$$

(5) $$\begin{array}{r} \frac{dw}{dt}=\Phi \left(w_{\infty }-w\right)/\tau_{w} \end{array}$$

where *C*_*m*_ is the specific membrane capacitance, *V* is the membrane potential, *w* is a slow recovery variable, and *I* is the stimulus applied to the neuron which has the same form with that used in the RGC model. *g*_*Na*_, *g*_*K*_, and *g*_*L*_ are the conductances for each currents. *V*_*Na*_, *V*_*K*_, and *V*_*L*_ are the reversal potentials. Φ is a scaling factor.

In Equations (4) and (5), *m*_∞_ = 0.5(1+tanh((*V* − β_*m*_)/γ_*m*_)), *w*_∞_ = 0.5(1 + tanh((*V* − β_*w*_)/γ_*w*_)), τ_*w*_ = 1/cosh((*V* − β_*w*_)/(2γ_*w*_)).

Other parameters used in simulations are shown in Table 1.

It should be noted that the ML model used in this study is to generate typical features of the three classes of excitability, and compare to the performance of RGC model, the ML model itself cannot generate the repetitive spiking, tonic firing and phasic firing behaviors of RGCs.

Simulations of the RGC and ML models were performed in the MATLAB environment (R2010a), and the fourth-order Runge-Kutta algorithm was employed to calculate the voltage values of neurons with time integration being 0.01 ms.

## Results

### Similar performance between the three types of RGC and the ML neurons

**Figure 1:**
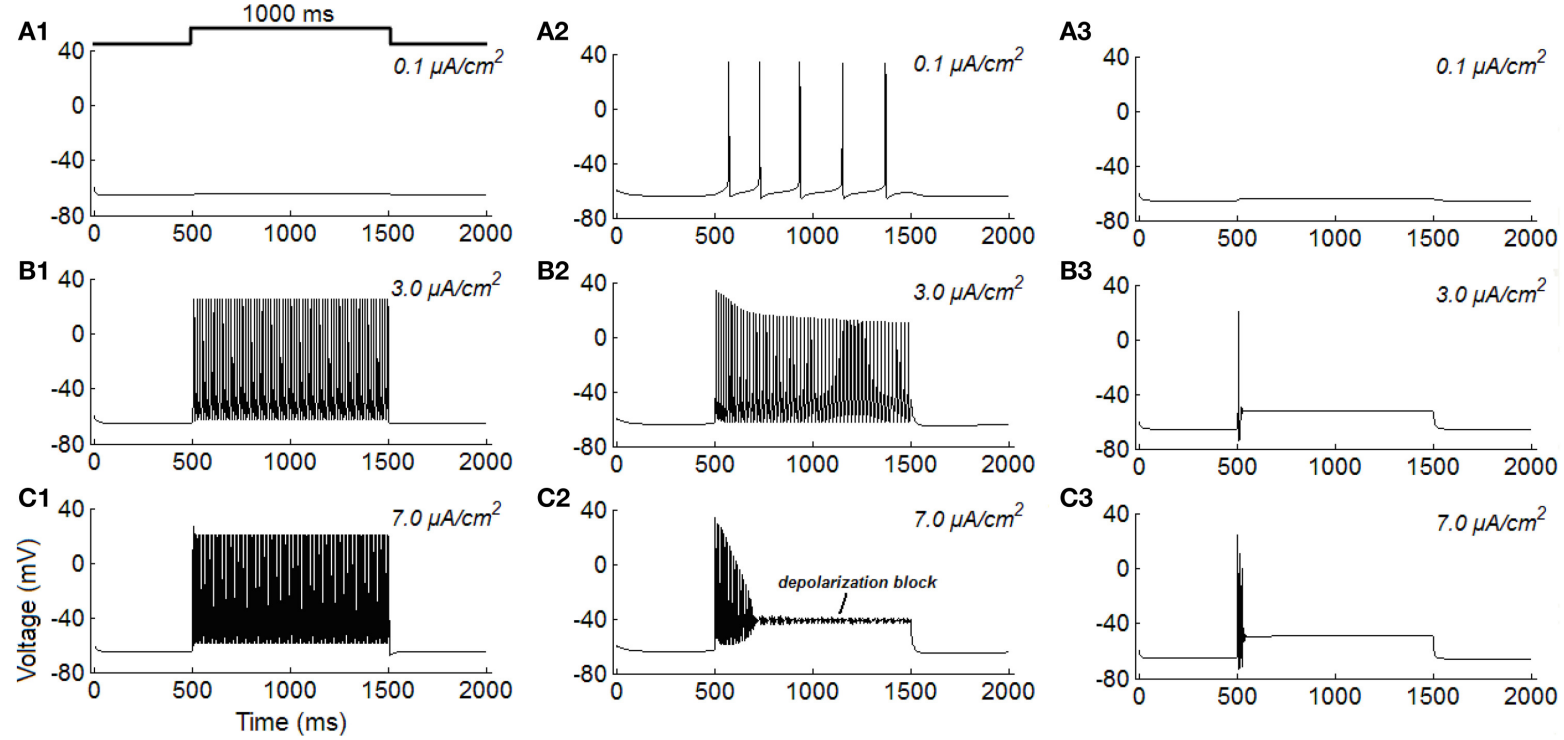
**Simulated repetitive spiking, tonic firing, and phasic firing activities of RGCs under constant current injections (unit: μ***A/cm***^**2**^)**. **(A1–C1)** Repetitive spiking; **(A2–C2)** Tonic firing; **(A3–C3)** Phasic firing.

Firstly, the repetitive spiking, tonic firing, and phasic firing behaviors of RGCs are simulated (Figure 1). It is clear that, the increase of stimulus intensity induces the repetitive spiking RGC switch from quiescent to fast spiking state, and the larger the stimulus intensity is, more spikes the neuron would elicit (Figures 1A1–C1), this variation trend is similar to that reported in Fohlmeister and Miller (1997), Fohlmeister et al. (2010) and Margolis and Detwiler (2007). For tonic RGC, it is found that the increase of stimulus intensity induces the neuron change from low-frequency firing state (Figure 1A2) to high-frequency firing state (Figure 1B2). However, when the stimulus intensity is too large, the neuron rapidly accommodates to a steady state in the late period of stimulation, a phenomenon called *depolarization block* (Figure 1C2). This variation trend is quite similar to previous experimental results (Kawai and Sterling, 2002; Tabata and Kano, 2002). While for phasic RGC, it is apparent that the increase of stimulus intensity makes the neuron transform from resting state (Figure 1A3) to spiking state at the onset of stimulus (Figures 1B3–C3). This variation trend is also similar to the experimental observations (Tabata and Kano, 2002; Kawai and Sterling, 2002).

In **Figure 3A**, it is clear that spike numbers of repetitive and phasic RGCs show similar monotonous increasing trends, with the spikes of repetitive spiking RGC being much more than those of the phasic RGC. However, spike numbers of tonic RGC demonstrate a non-monotonous variation trend: a high-frequency region is observed when the stimulus intensity locates in [1.5–5], while a low-frequency region is observed when the stimulus intensity locates in [6–8.5]. In the following analyses, we will separately discuss the response patterns of RGCs when stimulus intensity locates in these two different regions.

As observed in **Figure 3A**, the frequency of a repetitive spiking RGC varies in a discontinuous manner under weak stimulus, while the frequency of a tonic RGC varies in a continuous way. These variation trends are similar to that observed in Class I and Class II neurons. Moreover, the frequency of phasic RGC always stays in low states, which is also similar to the property of Class III neuron.

**Figure 2:**
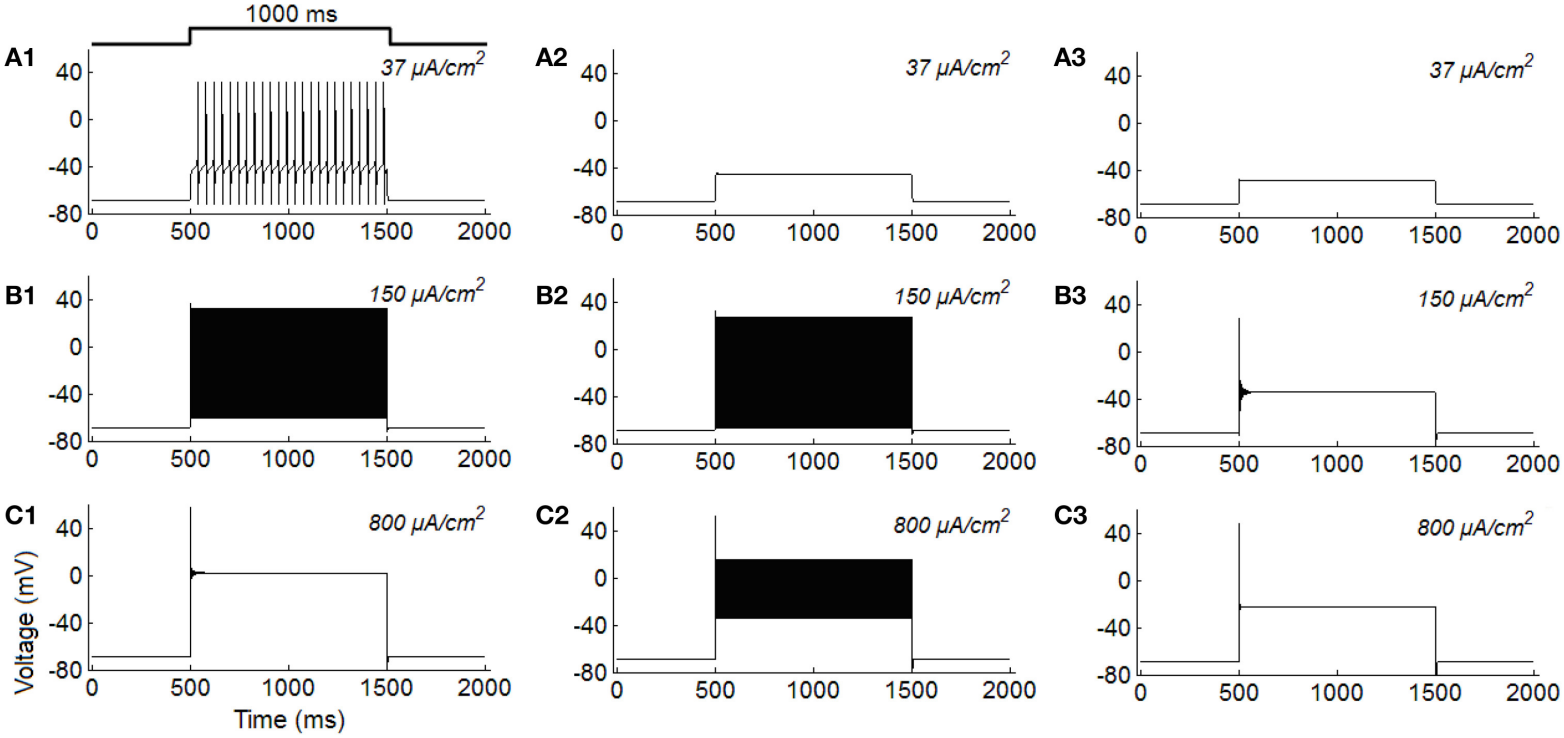
**Class I, class II, and class III firing behaviors simulated in the ML model under constant current injections (unit: μ***A/cm***^**2**^). (A1–C1)** Class I excitability; **(A2–C2)** Class II excitability; **(A3–C3)** Class III excitability.

To make a better comparison, we simulate typical features of the three classes of excitability using the ML model. Results in Figure 2 are the typical firing behaviors of the three classes of neurons under different stimulus intensities. It is shown that the overall variations of firing activity of the ML neurons are comparable to the results observed in RGCs (Figure 1).

**Figure 3:**
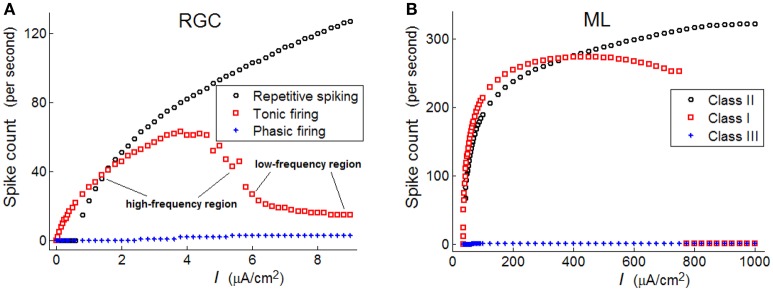
**Spike counts of the three types of RGC and the three classes of ML neurons with respect to stimulus intensity (unit: μ***A/cm***^**2**^)**. **(A)** RGCs; **(B)** ML neurons.

In Figure 3B, spike counts of the three neurons (ML) with respect to stimulus intensity are provided, which is clear that Class I neuron shows a continuous trend under weak stimulus, Class II neuron shows a discontinuous trend under weak stimulus, while Class III neuron always fires in low frequency states. Comparing Figure 3A with Figure 3B, we may conclude that repetitive spiking RGC exhibit Class II excitability, tonic RGC exhibit Class I excitability, and phasic RGC exhibit Class III excitability.

**Figure 4:**
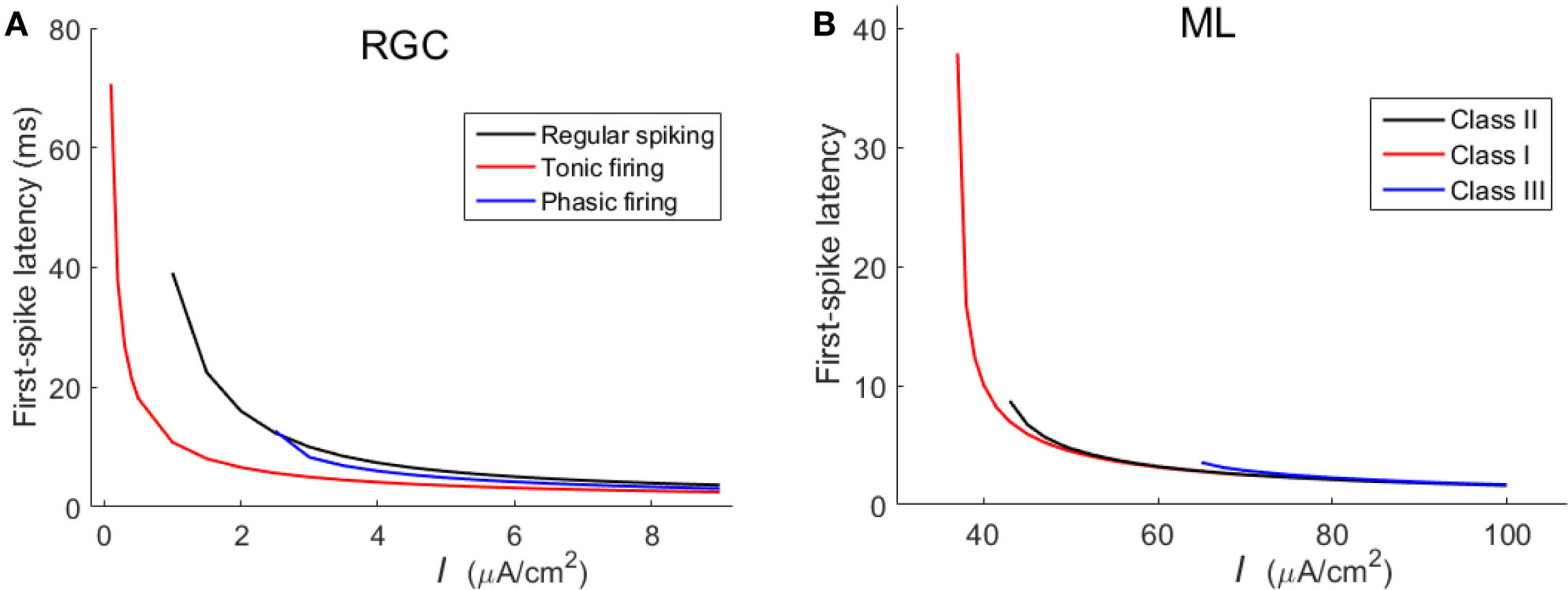
**First-spike latency of the three types of RGC and the three classes of ML neurons with respect to stimulus intensity (unit: μ***A/cm***^**2**^). (A)** RGCs; **(B)** ML neurons.

In addition to the spike counts, we also compare the spike latencies between the three RGCs and the three ML neurons. Results in Figure 4 demonstrate that RGCs exhibit much similar variation trends of latency with that of ML neurons, which further confirm that the three RGCs can exhibit Hodgkin's three classes of excitability.

### Amplitude-dependent firing characteristics of RGCs under periodic stimuli

Next, the capabilities of RGCs in encoding periodic signals are investigated. Two coding measures which have been widely used to characterize neural signals are employed, one measure is the firing rate (spikes/cycle), which was chosen following the studies from van Brederode and Berger (2008) and Descalzo et al. (2005). We also did simulations using spikes/second to measure firing rate, and obtained similar comparison results between the RGC model and ML model (see Supplementary Figure); Another measure is the first-spike latency. The reason we adopt these two measures is that firing rates and first-spike latency have both found to effectively encode visual information in RGCs (van Rullen and Thorpe, 2001; Gollisch and Meister, 2008; Gütig et al., 2013).

**Figure 5:**
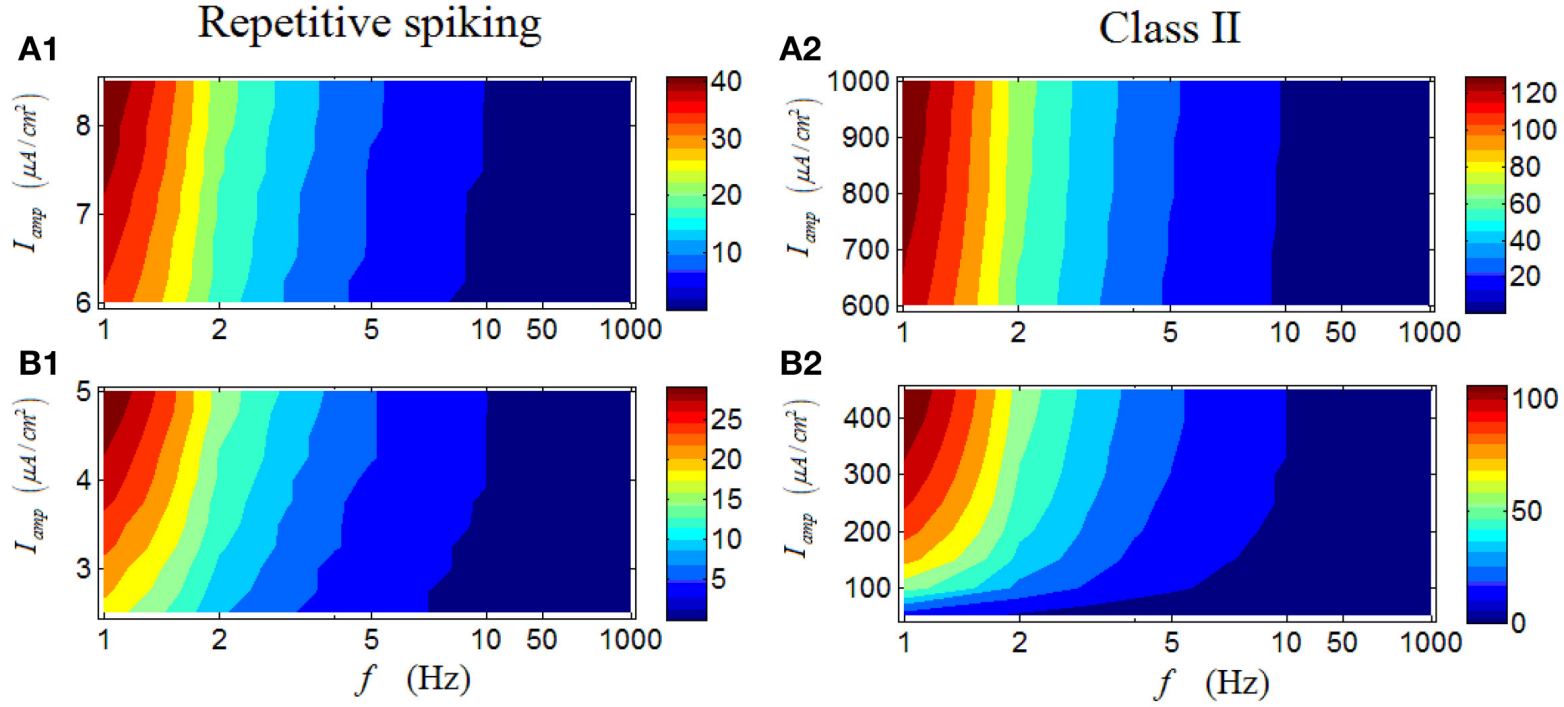
**Firing rates (spikes/cycle) of a repetitive spiking RGC and a Class II neuron under periodic stimuli. (A1,A2)** Firing rates under high stimulus amplitude (*I*_*amp*_). **(B1,B2)** Firing rates under low stimulus amplitude (*I*_*amp*_).

As shown in Figure 5A1, under high *I*_*amp*_, there is an obvious region in which the firing rate of a repetitive spiking RGC with large values are located (dark red area), primarily under low stimulus frequencies. When low *I*_*amp*_ is applied, the phenomenon maintains, with only minor differences (Figure 5B1). These results suggest that repetitive spiking RGCs activate strongly under low stimulus frequencies, larger frequency would lower their firing activity. Moreover, the variation of *I*_*amp*_ barely alter this phenomenon. Figures 5A2–B2 demonstrates the results from a Class II neuron, which clearly show the similarity with those in a repetitive RGC.

**Figure 6:**
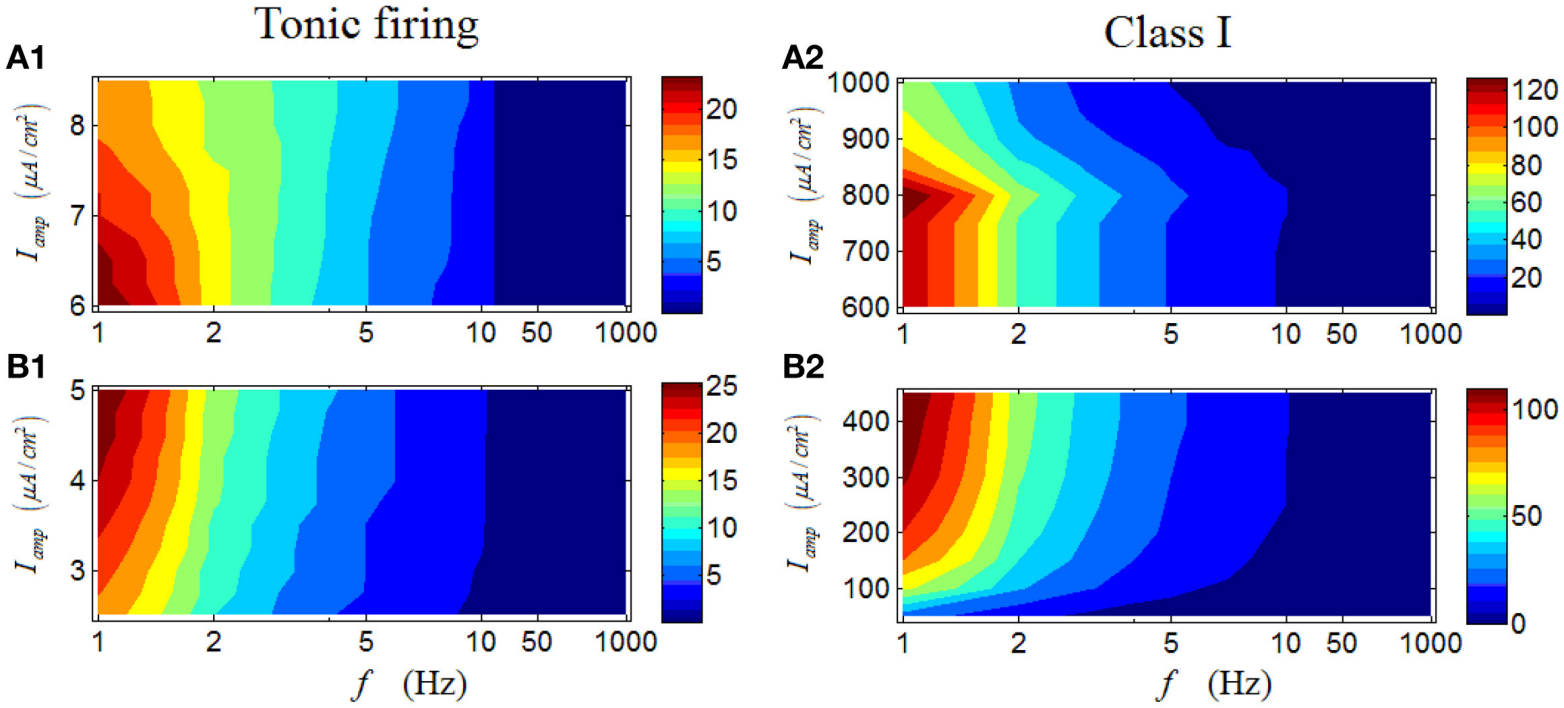
**Firing rates (spikes/cycle) of a tonic RGC and a Class I neuron under periodic stimuli. (A1,A2)** Firing rates under high stimulus amplitude (*I*_*amp*_). **(B1,B2)** Firing rates under low stimulus amplitude (*I*_*amp*_).

Results in Figures 6A1–B1 are the case for a tonic RGC. It is clear that under high *I*_*amp*_, there is also an obvious region in which the firing rate with large values are located (dark red area in Figure 6A1), mainly under low stimulus frequencies. When low *I*_*amp*_ is applied, the observed feature persists (Figure 6B1), but a small distinction still can be seen. Under low *I*_*amp*_, the increase of stimulus amplitude would enhance the firing rate; while under high *I*_*amp*_, the increase of stimulus amplitude would weaken the firing rate, comparable with the result in Figure 3. These results indicate that although the variation of stimulus amplitude will not alter the general response patterns of a tonic RGC, its detailed modulations are totally opposite when stimulus amplitude varies in the two different regions. Similar phenomenon can be observed in a Class I neuron (Figures 6A2–B2).

**Figure 7:**
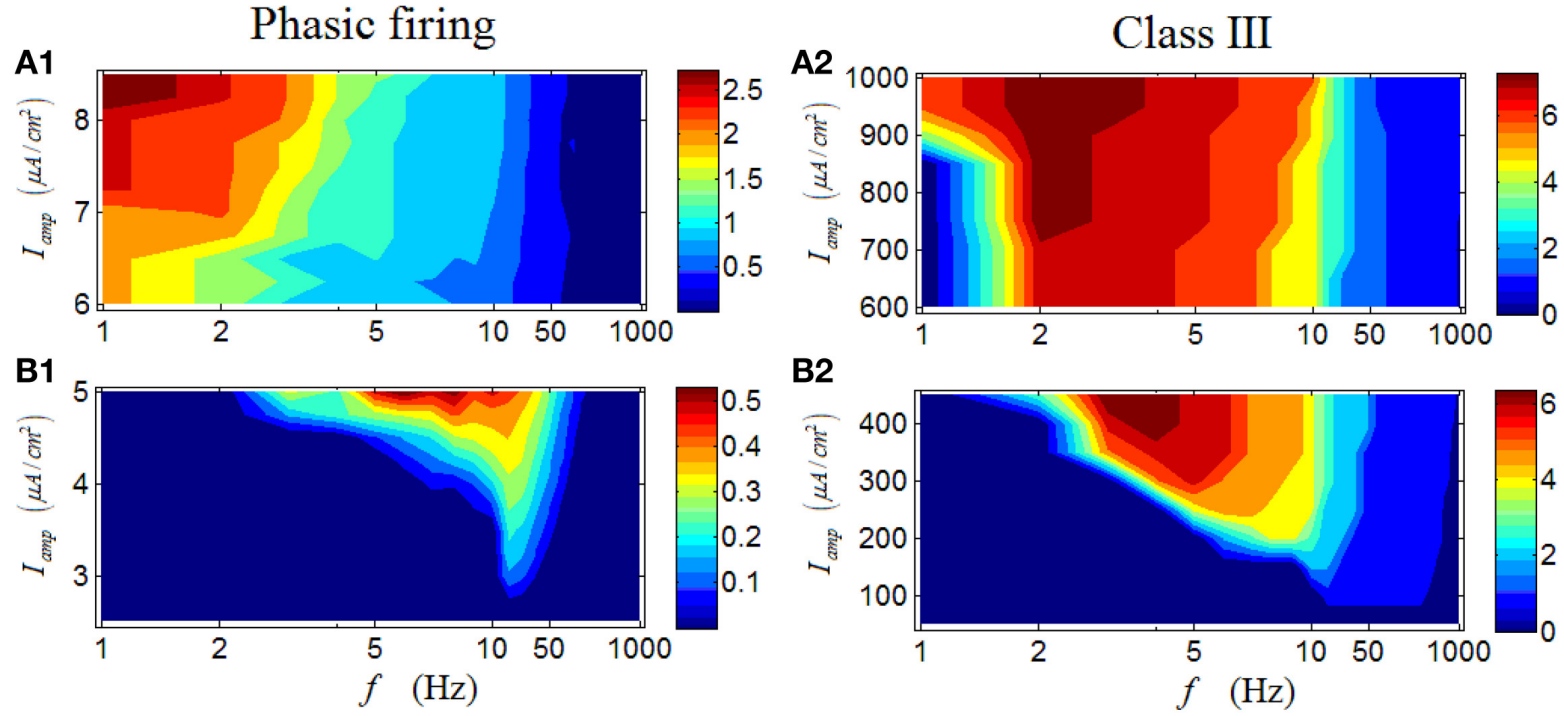
**Firing rates (spikes/cycle) of a phasic RGC and a Class III neuron under periodic stimuli. (A1,A2)** Firing rates under high stimulus amplitude (*I*_*amp*_). **(B1,B2)** Firing rates under low stimulus amplitude (*I*_*amp*_).

Results for a phasic RGC are illustrated in Figures 7A1–B1. It is apparent that the response characteristics are totally different between high and low *I*_*amp*_. Under low *I*_*amp*_, the phasic RGC responds vigorously at medium levels of stimulus frequency (Figure 7A1); while under high *I*_*amp*_, the phasic RGC switches to activate strongly at low stimulus frequencies (Figure 7B1). This group of result reveals that a phasic RGC can perform two different spike-rate coding operations, depending on the stimulus amplitude. Similar conclusion can be drawn in a Class III neuron (Figures 7A2–B2).

**Figure 8:**
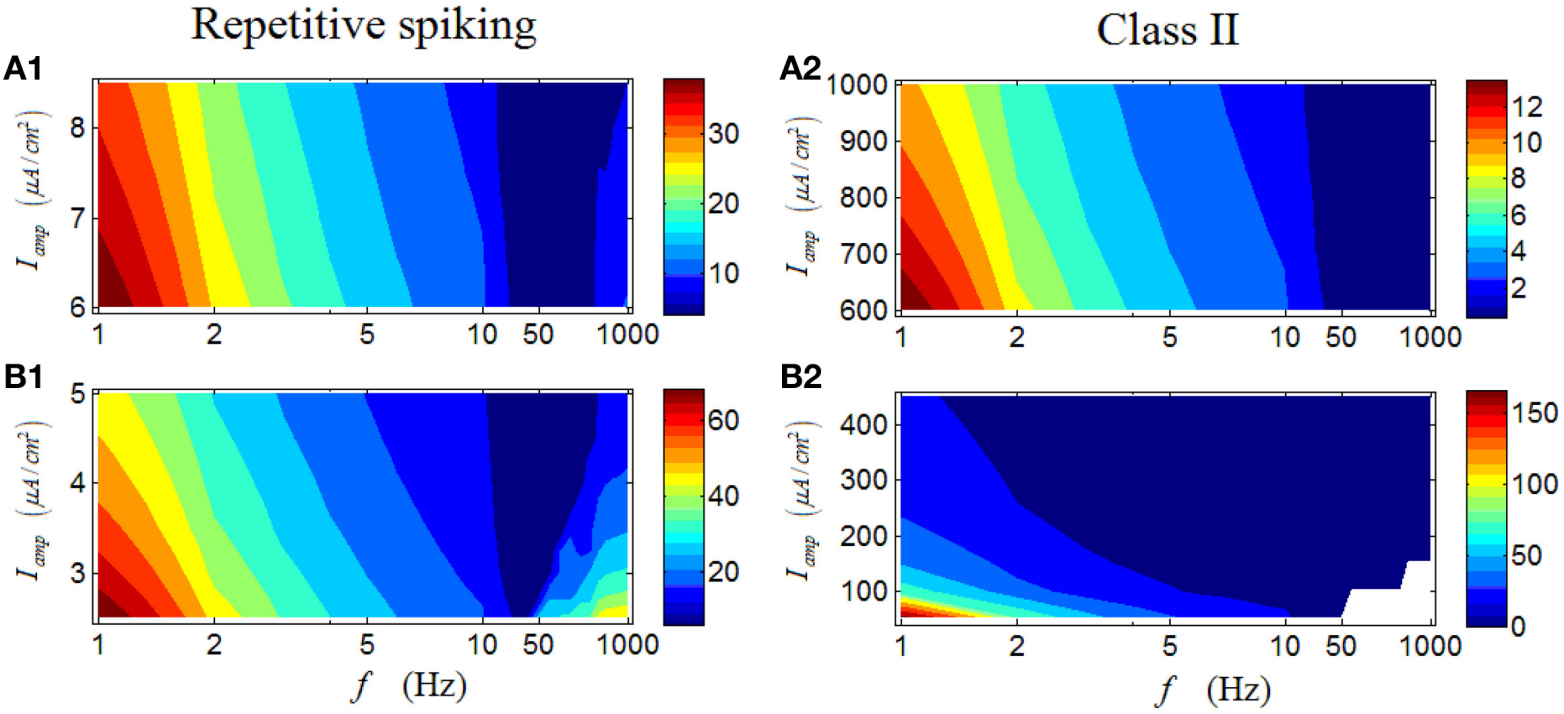
**First-spike latency of a repetitive spiking RGC and a Class II neuron under periodic stimuli. (A1,A2)** First-spike latency under high stimulus amplitude (*I*_*amp*_). **(B1,B2)** First-spike latency under low stimulus amplitude (*I*_*amp*_). White area means there is no response of the neuron.

In the followings, we continue our study by analyzing the first-spike latency of RGCs. Results shown in Figures 8A1–B1 are the case for a repetitive spiking RGC, which indicate that the first-spike latency with large values are mostly located in a region with low stimulus frequencies (dark red area in Figures 8A1–B1). Meanwhile, variation of the stimulus amplitude has little influence on the general response pattern of the RGC. This result suggests that for a repetitive spiking RGC, relative high stimulus frequency would lead the neuron respond more rapidly, consistent with the result in Wang et al. (2013). Similar phenomenon can be observed in a Class II neuron (Figures 8A2–B2).

**Figure 9:**
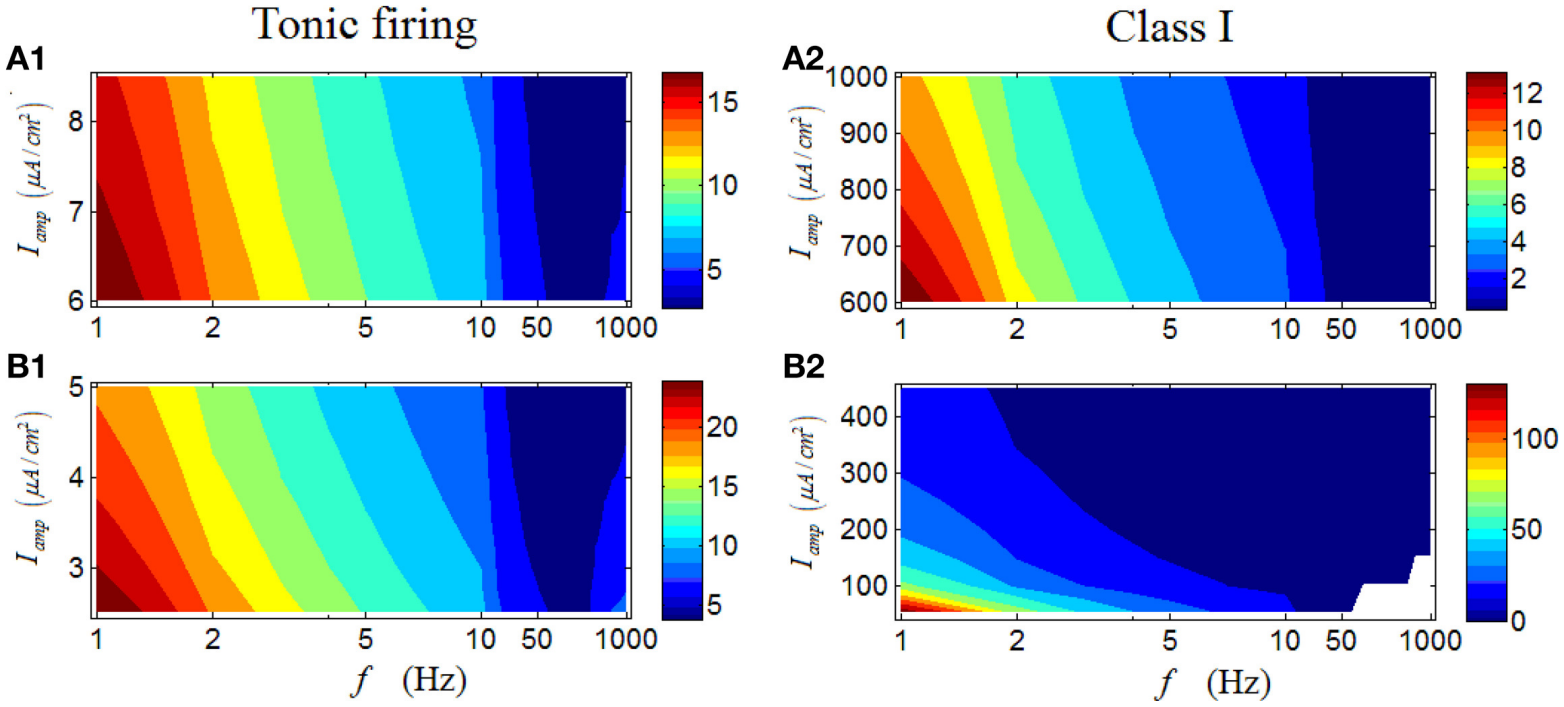
**First-spike latency of a tonic RGC and a Class I neuron under periodic stimulus. (A1,A2)** First-spike latency under high stimulus amplitude (*I*_*amp*_). **(B1,B2)** First-spike latency under low stimulus amplitude (*I*_*amp*_). White area means there is no response of the neuron.

For a tonic RGC, its first-spike latency shows a similar variation trend with that of a repetitive spiking RGC (Figures 9A1–B1). However, unlike the results in Figure 6, in which the change of stimulus amplitude can markedly affect the firing rate of a tonic RGC, stimulus amplitude has little influence on the patterns of first- spike latency of the tonic RGC. Similar results can be produced by a Class I neuron (Figures 9A2–B2).

**Figure 10:**
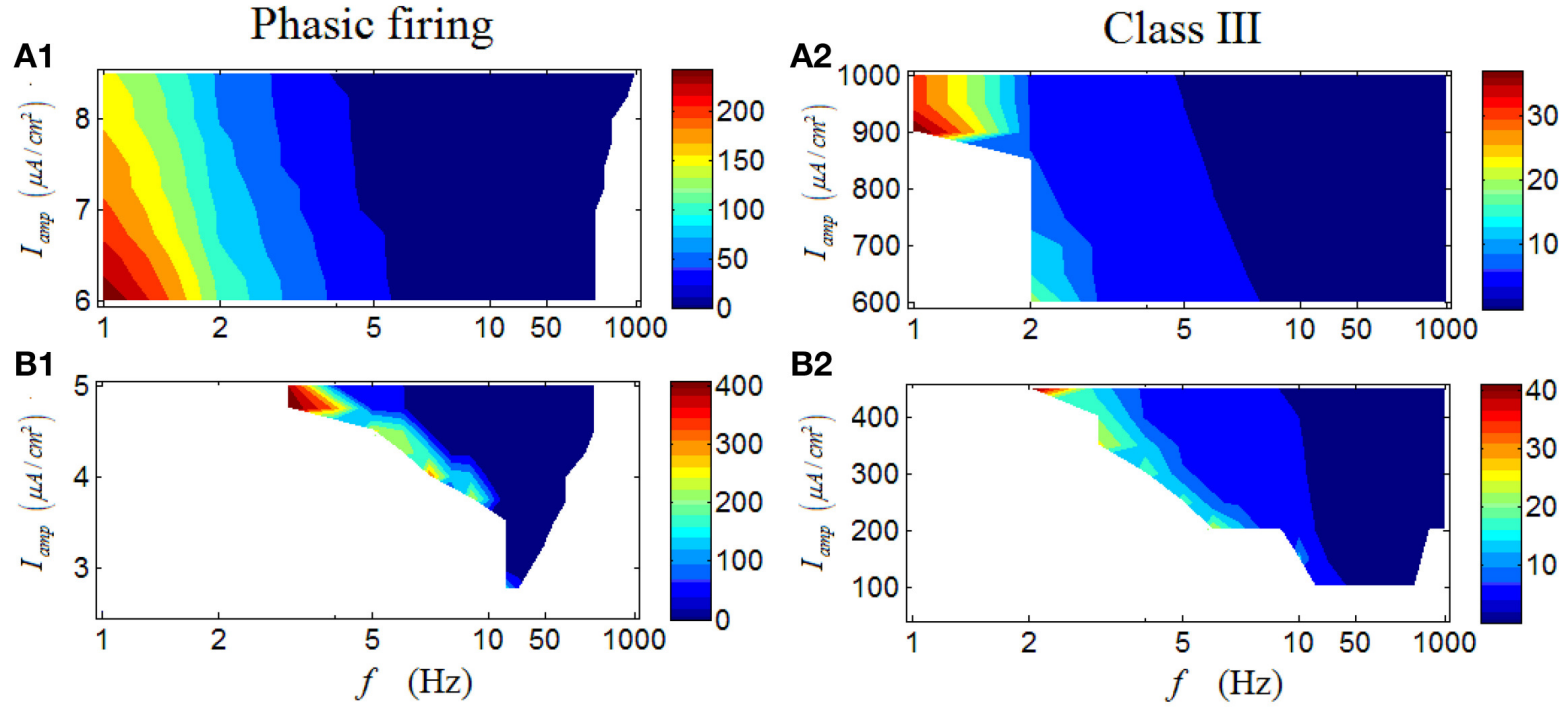
**First-spike latency of a phasic RGC and a Class III neuron under periodic stimuli. (A1,A2)** First-spike latency under high stimulus amplitude (*I*_*amp*_). **(B1,B2)** First-spike latency under low stimulus amplitude (*I*_*amp*_). White area means there is no response of the neuron.

However, unlike the case for a repetitive spiking and a tonic RGC, the latency of a phasic RGC exhibits rather different variation trends. As demonstrated in Figure 10B1, a valley can be observed when *I*_*amp*_ is low, in which the latency decreases with the increase of stimulus frequency. When *I*_*amp*_ is high, the variation of latency with respect to stimulus frequency begins to change, specifically, the latency also decreases with the increased stimulus frequency but with a wider frequency range (Figure 10A1). In short, this group of results indicates that phasic RGC can perform a stable spike-latency coding pattern, and the change of stimulus amplitude would alter the response frequency range but would not alter this pattern significantly. A Class III neuron can exhibit similar variation trends (Figures 10A2–B2).

## Discussions

By revealing the exact mechanisms of various firing patterns in neurons, one can gain insight to different spiking behaviors and the nature of transition modes between them. This understanding is quite inspirational in promoting the exploration of how different factors might influence neural firing patterns. In this study, we performed a model investigation on the response properties of repetitive spiking, tonic firing, and phasic firing RGCs under periodic stimuli. Two coding measures (spike-rate and spike-latency) are employed to characterize the capability of the three RGCs in encoding periodic signals. The presented results suggest that repetitive spiking, tonic firing, and phasic firing RGCs can exhibit different firing characteristics in exposure to periodic stimuli. Meanwhile, our results further reveal that the dynamic response properties of RGCs are sensitive to the stimulus amplitude. More importantly, the results observed from the three RGCs can be well reproduced by Hodgkin's three classes of neuron (ML model).

As the first information processing center in the visual pathways, RGCs undertake important functions in encoding the incoming visual signals. However, a frequently encountered problem for biological neurons is that their signaling capacity is too limited. Thus, to effectively encode the vast amount of visual inputs, RGCs must selectively react to some aspects of input signals, and ignore other aspects. Model results in this study suggest that different RGCs can perform different operations in encoding periodic signals. Repetitive spiking and tonic RGCs prefer to activate strongly under low-frequency stimulus, but respond weakly to high- frequency stimulus. On the other hand, phasic RGCs activate vigorously under medium levels of frequency, but respond sluggishly to low- and high-frequency stimuli. These distinctions may indicate that RGCs can expand their dynamic range through complementary actions. A similar phenomenon has been observed in frog second-order vestibular neurons (2°VN) (Beraneck and Straka, 2011), in which the authors suggested that frog tonic 2°VN prefer to respond strongly to low-frequency stimulus, like low-pass filters, whereas phasic 2°VN prefer to respond strongly at medium levels of stimulus frequency, like band-pass filters.

The transformation of incoming signals into spikes by neurons is recognized to be the basis for neural information processing (neural coding). During the past decades, several efficient coding strategies have been proposed, such as: rate coding (Descalzo et al., 2005; van Brederode and Berger, 2008), and time coding (Mainen and Sejnowski, 1995; Chase and Young, 2007; Zhang et al., 2010). A recent study suggested that RGCs can encode input stimulus into both spike-rate and spike-latency simultaneously (Xiao et al., 2014). In this study, we further suggest that RGCs with intrinsically heterogeneities may encode periodic stimuli into different spike-rate patterns and spike-latency patterns; moreover, the patterns of spike-rate and spike-latency produced by the same RGCs are quite similar.

Excitability is an extraordinary feature in many biological neurons. Traditionally, neuronal excitabilities can be categorized into three basic classes (I, II, III) based on their electrophysiological responses to constant current injections (Hodgkin, 1948; Izhikevich, 2007). Many types of neurons have been found to exhibit part(s) or all of the three classes of excitability, for instance, some neurons were found to exhibit Class I or Class II firing dynamics (Tateno et al., 2004; Tateno and Robinson, 2006), or both Class I and II firing dynamics (Prescott et al., 2008b; Zeberg et al., 2010), while other neurons were observed to perform all the three classes of excitability (Prescott et al., 2008a). It has been reported that some ion channels exhibit crucial roles in distinguishing different classes of neurons, e.g., Na-K channel density (Johansson and Arhem, 1992; Arhem et al., 2006; Arhem and Blomberg, 2007), and M-type potassium current (Prescott et al., 2008b). In this model study, we suggest that RGCs can produce all the three classes of excitability by adjusting several ion channel densities. This is important, since RGCs play pivotal functions in encoding visual signals, to process the vast amount of light inputs efficiently, RGCs must adopt complementary strategies to expand their excitation range. Due to the universality of the three classes of excitability in neuronal systems, the results we observed from the RGCs model may be not specific, and we further infer that the results can applicable to other neurons in the brain regions which show part(s) or all of the three firing dynamics.

Previous reports have revealed that response patterns of periodically forced neurons can be modulated by a variety of factors, e.g., intrinsic ion channels (Cangiano et al., 2007; Kamiyama et al., 2009) and active dendrites (Zhuchkova et al., 2013). Besides, our results suggest that in RGCs, stimulus amplitude is also a critical factor in modulating the capability of RGCs in encoding periodic signals. In summary, model results in this study may provide new insights in further understanding how RGCs encode periodic afferents.

It should be noted that the model we analyze in this study is a simplified single-compartment neuron model with only one soma and did not include any dendritic information. During the past decades, a great number of studies adopt single-compartment models as basis to simulate firing behavior of biological neurons and neuronal systems. However, it is still insufficient and inaccurate, since dendrites also play important roles in regulating neuronal activities. Thus, one extension of our study may concentrate on using more detailed models with active dendrites to simulate the firing behavior of RGCs using periodic forces. In addition, RGCs are more prone to activate collectively than activate independently, previous researches have suggested that gap junctions are abundant between RGCs, also between RGCs and other retinal cells (Völgyi et al., 2013). So, another extension of our study may concentrate on networks of RGCs coupled with gap junctions, and investigate how periodic forces would influence the network activity of RGCs.

## Author contributions

LW and YQ designed and wrote this paper, LW collected and analyzed the data, YQ and YZ revised it and approved it.

### Conflict of interest statement

The authors declare that the research was conducted in the absence of any commercial or financial relationships that could be construed as a potential conflict of interest.
