# Supplementary figures and images for: Coding Properties of Three Intrinsically Distinct Retinal Ganglion Cells under Periodic Stimuli: A Computational Study
Source: Front Comput Neurosci. 2016 Sep 23;10:102. doi: 10.3389/fncom.2016.00102 (PMC5033956; doi:10.3389/fncom.2016.00102)

## Supplementary figures

Firing rate measure using spikes/second

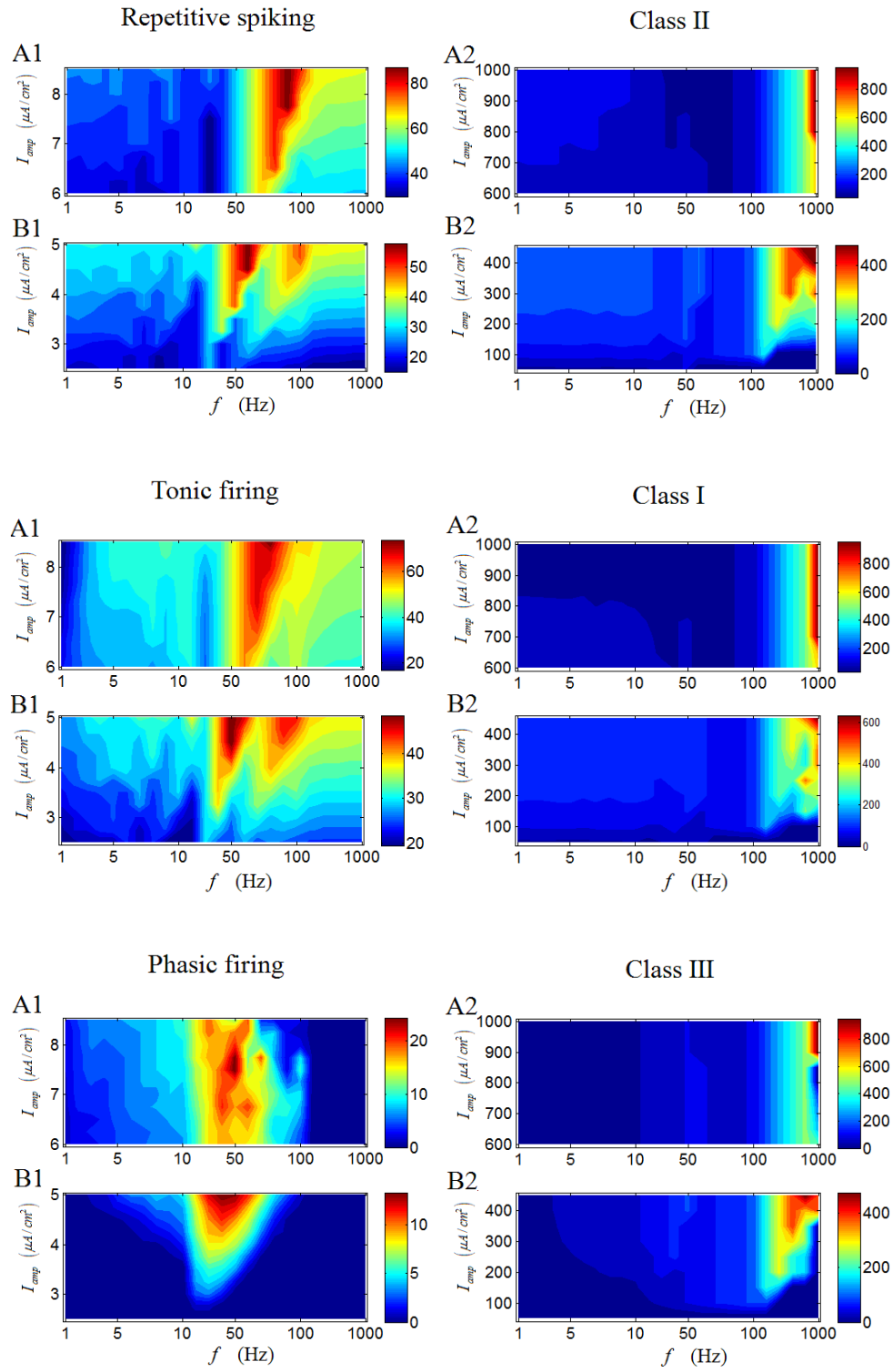

Supplement: Supplementary file 1 [file Image1.PDF]
